# High-resolution and sensitivity bi-directional x-ray phase contrast imaging using 2D Talbot array illuminators

**DOI:** 10.1364/OPTICA.441004

**Published:** 2021-12-10

**Authors:** Alex Gustschin, Mirko Riedel, Kirsten Taphorn, Christian Petrich, Wolfgang Gottwald, Wolfgang Noichl, Madleen Busse, Sheila E. Francis, Felix Beckmann, Jörg U. Hammel, Julian Moosmann, Pierre Thibault, Julia Herzen

**Affiliations:** 1 Department of Physics and Munich School of Bioengineering, Technical University of Munich, 85748, Garching, Germany; 2 Institute of Materials Physics, Helmholtz-Zentrum Hereon, Max-Planck-Str. 1, 21502 Geesthacht, Germany; 3 Department of Infection, Immunity and Cardiovascular Disease, Medical School, University of Sheffield S10 2RX, UK; 4 Department of Physics, University of Trieste, Trieste 34217, Italy

## Abstract

Two-dimensional (2D) Talbot array illuminators (TAIs) were designed, fabricated, and evaluated for high-resolution high-contrast x-ray phase imaging of soft tissue at 10–20 keV. The TAIs create intensity modulations with a high compression ratio on the micrometer scale at short propagation distances. Their performance was compared with various other wavefront markers in terms of period, visibility, flux efficiency, and flexibility to be adapted for limited beam coherence and detector resolution. Differential x-ray phase contrast and dark-field imaging were demonstrated with a one-dimensional, linear phase stepping approach yielding 2D phase sensitivity using unified modulated pattern analysis (UMPA) for phase retrieval. The method was employed for x-ray phase computed tomography reaching a resolution of 3 µm on an unstained murine artery. It opens new possibilities for three-dimensional, non-destructive, and quantitative imaging of soft matter such as virtual histology. The phase modulators can also be used for various other x-ray applications such as dynamic phase imaging, super-resolution structured illumination microscopy, or wavefront sensing.

## 1. INTRODUCTION

Various imaging techniques based on x-rays have opened unique insights into three-dimensional (3D) structures at the micro- and nanometer scale and even enabled the capture of time-resolved volumetric data due to recent innovations in x-ray sources, optics, detectors, high precision metrology, and advanced post-processing and reconstruction algorithms. Phase contrast techniques have become indispensable due to their capability to generate superior contrast in soft tissue compared to conventional attenuation-based mechanisms [1]. While propagation-based methods provide good edge visibility [2], analyzer-based [3], interferometric [4,5], aperture-based [6,7], and speckle-based methods [8–10] enable us to retrieve the attenuation, phase, and dark-field signals separately from a measurement at one single propagation distance. The latter techniques rely on various diffractive and absorptive beam modulator optics creating a defined intensity pattern after propagation in space. This modulation is altered by absorption, refraction, and scattering by the investigated object in the beam path. Various techniques have been successfully implemented to retrieve those different interactions from a sample and a reference scan both in single-shot mode [9–12] and from multiple exposures with stepped modulators [8,13–15]. In speckle-based imaging (SBI), a random modulation is introduced by a diffuser (e.g., sandpaper with a fine grain size), while other techniques generate periodic modulations with gratings or other diffractive or refractive arrays. In order to perform efficiently, the modulators have to generate a pattern with good contrast (visibility) and average feature sizes resolvable by the detector.

In general, a stable and high-resolution bi-directional phase retrieval requires every detector pixel to undergo a high contrast modulation in both directions during phase stepping. For random speckle patterns, this requires a large number of stepped frames at the cost of longer acquisition times, higher radiation dose, and complexity in data handling and image processing [15]. Using a regular beam modulator (e.g., a grating pattern or a refractive lens array) and applying an adapted sampling scheme can avoid these problems and reduce the number of frames required to reach a high resolution and sensitivity.

Currently, a remarkable effort is being undertaken to create such periodic diffractive optical elements (DOE) for a variety of x-ray applications. Reich *et al*. [16] created an array of stacked compound refractive lenses (CRLs) with a period of 65 µm. Dos Santos Rolo *et al*. [17] fabricated a Shack–Hartman array with 20×20 micro-lenslets by 3D direct laser writing with a periodicity of 50 µm. Kagias *et al*. [18] fabricated circular phase arrays for omnidirectional dark-field imaging with a unit cell period of 80 µm. Mamyrbayev *et al*. [19] developed a two-dimensional (2D) CRL array for sub-pixel resolution scanning transmission microscopy with a period of 55 µm. As some of these recent examples show, the periods of such x-ray optics are still in the range of several tens of micrometers limiting the achievable performance in sensitivity and resolution. Different types of 2D gratings [7,12,20–24] have been used with significantly smaller periods. However, they did not achieve comparable visibilities and flux efficiencies as the aforementioned beam modulators, which create periodic sharp foci in the detection plane.

Optimized modulators should have a high x-ray transmission (to be flux-efficient) and a strong resistance to high radiation doses. Furthermore, they should be easy to fabricate with current microprocessing technologies and adaptable in period in the sub-10-µm range to operate at high visibility with a given detector point spread function (PSF). Considering these factors, we propose and demonstrate a 2D periodic phase-shifting grating for the x-ray regime, also known as Talbot array illuminator (TAI) from visible light literature [25–27]. Compared to previously described methods employing 2D phase gratings [12,21,22,28], we have adapted a design that creates periodic foci with a higher compression ratio compared to, e.g., checkerboard 2D modulators [12] or orthogonally stacked one-dimensional (1D) linear gratings [28]. In contrast to absorptive 2D gratings or Hartmann masks previously demonstrated for x-rays [7,29,30], the proposed TAI uses the entire transmitting radiation to generate the desired modulation. Compared to state-of-the-art refractive micro-lens arrays [16,17,31], the fabricated phase arrays have a much larger field-of-view (FoV), are resistant to long and high radiation dose exposures, and can be easily fabricated with up to 1 order of magnitude smaller periods (e.g., 5 µm). Unlike random phase modulators (diffusers) used in SBI, the TAI can be tailored for optimal performance at a certain source coherence, spectral range, propagation distance, and detector PSF. All of these aspects become crucial when the method is translated from coherent sources at large synchrotron facilities to laboratory-based micro-focus sources with polychromatic spectra.

In the present research, we evaluate customary designed TAIs of different periods and compare their visibility performance with a sandpaper diffuser at different propagation distances. Further, a 1D stepping scheme yielding bi-directional sensitivity is employed and compared with the random modulator for different numbers of phase steps. High-resolution bi-directional phase and dark-field imaging are demonstrated, and a computed tomography (CT) phase scan of a murine artery embedded in paraffin is acquired. The proposed TAIs and acquisition schemes facilitate current state-of-the-art x-ray phase tomography, providing a convenient pathway for non-destructive, quantitative high-resolution 3D virtual histology.

## 2. DESIGN OF TALBOT ARRAY ILLUMINATORS

**Fig. 1.**
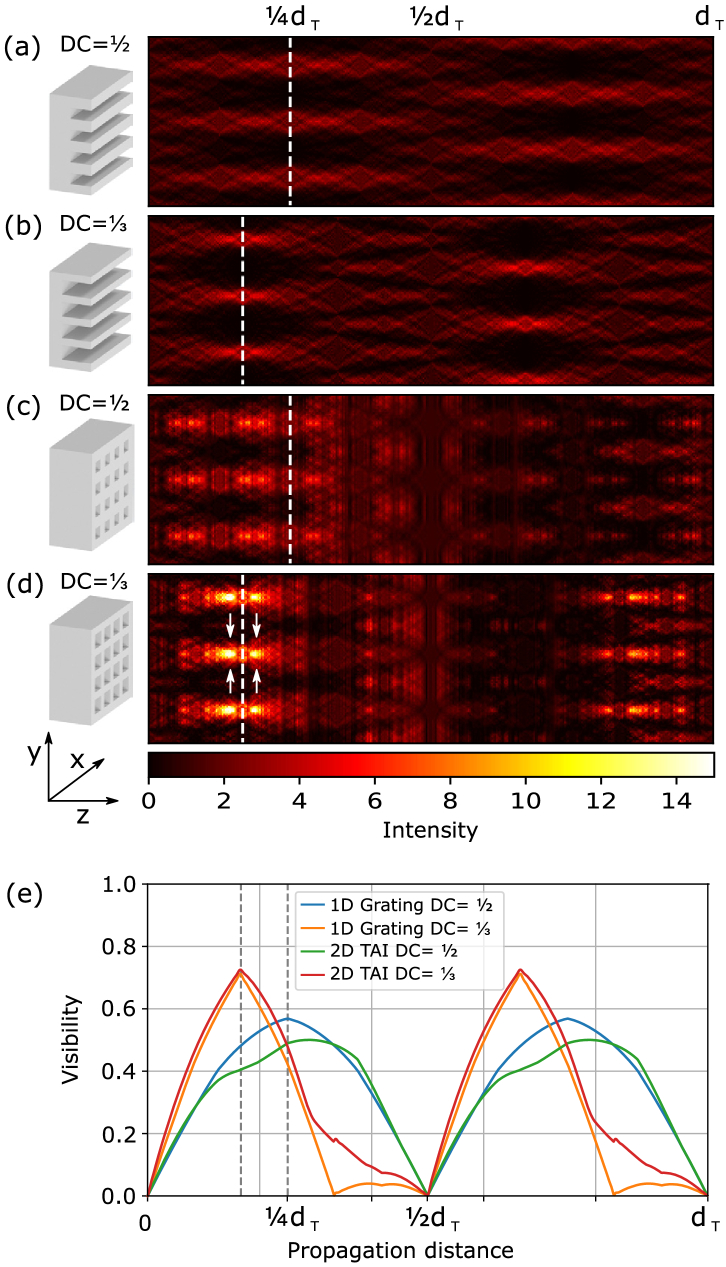
Simulated Talbot carpets for (a) 1D linear grating with symmetric duty cycle (DC) and phase shift φ=π/2 , (b) 1D linear grating with DC=1/3 and φ=2π/3 , (c) 2D TAI with DC=1/2 and φ=π/2 , and (d) 2D TAI with DC=1/3 and φ=2π/3 . The intensity is normalized to 1 in the grating plane. (e) Visibility with increasing propagation distance for the gratings (a)–(d). The fractional Talbot distances at which the first binary intensity modulation occur are denoted by dashed lines. (c) and (d) In the case of the 2D TAIs, the intensity modulation at $3d_{T}/4$ and $2d_{T}/3$ is not visible as it is shifted by half-period out of the plotted plane in the x direction.

**Fig. 2.**
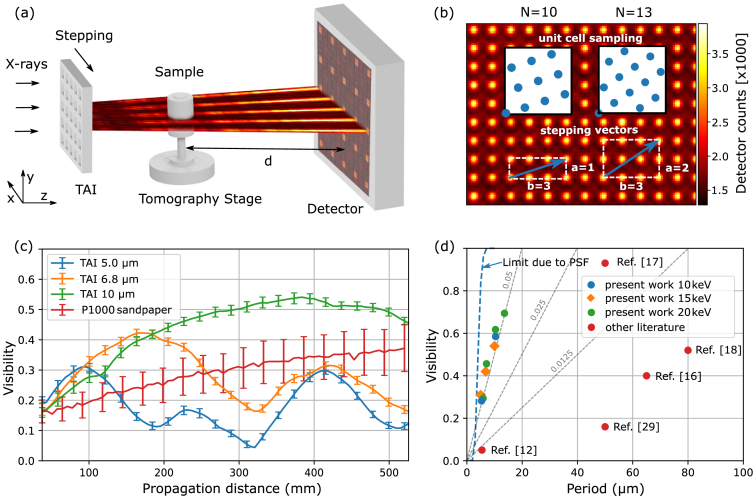
(a) General setup of the imaging system illustrating the formation of the intensity modulation. (b) Measured intensity pattern with a high compression ratio in the background and illustration of the proposed 1D stepping scheme. The stepping vectors (blue arrows) represent the range and direction of the phase stepping. The sampling of the unit cell is exemplarily shown for both stepping vectors with N=10 and N=13 steps. (c) Distance-dependent visibility and its standard deviation (error bars every four points) for the TAIs and the P1000 diffuser extracted from the Talbot carpet scans at 15 keV. (d) Comparison of the evaluated TAIs in terms of visibility and period with recent literature. The dashed gray lines depict visibility-to-period ratios.

Current high-resolution x-ray detectors are thin scintillator screens focused with magnifying optics and coupled to CCD or CMOS pixel sensors, providing effective pixel sizes below 1 µm and a spatial resolution in the range of 1–2 µm. The task of creating the most efficient modulator consists of finding an optimal trade-off between the smallest possible period and the highest intensity contrast achievable with the PSF of the used detector. At the same time, the optics should attenuate the beam as little as possible, which makes phase arrays fabricated from thin, x-ray transparent materials such as silicon the first choice. A broad variety of such periodic phase modulators has been studied [25–27] analytically to predict binary modulations at certain fractions of the Talbot distance $d_{T}=2p^{2}/\lambda$ , where p is the period of the array and λ is the wavelength of the radiation. The highest theoretically achievable binary modulation with binary (two height levels) 1D linear phase gratings has a compression ratio of 1:3 [25], i.e., the entire radiation is focused onto lines with a width of 1/3p . A high compression ratio directly results in high visibility defined by

(1) $$V=\frac{I_{max}-I_{min}}{I_{max}+I_{min}},$$

where $I_{\max}$ and $I_{\min}$ denote the maximal and minimal intensity within one modulation period. The measured visibility will be reduced by the detector blur, which can be modeled by a convolution of the propagated intensity distribution with the PSF of the detector. In order to compare the performance of different grating designs, we calculated the resulting intensity patterns using the Fresnel–Kirchhoff diffraction formula (see Supplement 1 for details). The so-called Talbot carpets plotted in Figs. 1(a)–1(d) show how the spatial intensity evolves with the propagation distance. For binary gratings, it depends mainly on the duty cycle DC (ratio of the phase-shifting fraction of the period) and the phase shift φ of the grating profile. In most literature employing 2D phase gratings with x-rays (e.g., [12,21,22,28]) symmetric duty cycles ( DC=0.5 ) were used, which also result in symmetric intensity modulation at fractional Talbot distances. However, a much stronger contrast can be achieved with asymmetric DC configurations when a convenient phase shift is chosen. Figures 1(a) and 1(b) show calculated Talbot carpets of 1D linear phase gratings illustrating this intensity focusing effect. While the grating with symmetric duty cycle [Fig. 1(a)] produces an intensity modulation with a compression ratio of 1:2 at $d_{T}/4$ , the grating with DC=1/3 and φ=2π/3 [Fig. 2(b)] shows a stronger focusing with a compression ratio of 1:3 at an even shorter propagation distance ( $d_{T}/6$ ). There are also several other asymmetric grating parameter configurations that create the same effect at different propagation distances [25].

A wave propagation with 2D arrays shows that this principle can be directly extended to a respective two-level 2D modulator creating a binary modulation with a compression ratio of 1:9. In Figs. 1(c) and 1(d), the Talbot carpets for the 2D TAIs with the respective duty cycle designs are shown. As visible from the intensity [note that Figs. 1(a)–(d) have the same intensity color map), the 2D modulators result in an overall stronger focusing; therefore, higher contrast is achieved compared to the 1D gratings. Further, the 2D TAI with DC=1/3 [Fig. 1(d)] shows a significantly higher intensity than its symmetric counterpart [Fig. 1(c)]. Besides that, even stronger modulations (depicted by arrows) before and after the binary modulation at $d_{T}/6$ are observable.

To quantify the visibility gain using the asymmetric gratings compared to the conventional ones, a plot of the visibility with propagation distance is provided in Fig. 1(e). The visibility values have been calculated by Eq. (1) after convolving the intensity pattern at each propagation distance with a Gaussian 2D Kernel of σ=0.2p (accounting for PSF). The plot shows that both 1D and 2D modulators perform similarly in terms of visibility, although the 2D TAIs reach a higher compression ratio. That is comprehensible, as 2D-focused spots are affected stronger by the PSF blur compared to 1D linear intensity distributions. However, the advantage of the asymmetric modulators, both in 1D and 2D cases, is visible. The asymmetric 2D TAI reaches about a 40% higher visibility than its symmetric counterpart at respective peak performance.

It is noteworthy that even stronger compression ratios can be achieved with binary phase arrays using DC<1/3 . Although the created intensity pattern will not be binary, most of the intensity will be still focused on very narrow points [26]. More complicated phase modulators, e.g., with more than two height levels and sub-periodic features [27] or other non-binary [32] (e.g., triangular, trapezoidal, or sinusoidal) DOEs can also create stronger focusing than conventional binary phase gratings. However, they are more difficult to fabricate on the sub-10-µm period scale for x-rays than binary TAIs discussed in this work. Furthermore, there is no benefit (in terms of visibility) in focusing on areas much smaller than the detector PSF. We conclude that the discussed 2D TAI design with DC=1/3 and φ=2π/3 is an efficient and easy-to-fabricate x-ray phase array, serving the purpose of high-resolution phase contrast and dark-field imaging.

## 3. EXPERIMENTAL

Multiple TAIs with periods of 5.0 µm, 6.8 µm, 10.0 µm, and 13.6 µm with different heights adapted for energies of 10 keV, 15 keV, and 20 keV were fabricated and evaluated for their diffractive properties using coherent x-ray synchrotron radiation at the P05 imaging beamline [33,34] operated by Helmholtz-Zentrum Hereon at PETRA III at Deutsches Elektronen-Synchrotron (DESY), Hamburg, Germany. More details about the fabrication, setup parameters, and data processing are provided in Supplement 1. The general setup is shown in Fig. 2(a), where incoming x-rays are modulated by the TAI, interact with the sample, and are then recorded by the detector. First, Talbot carpets [indicated by the colored layers in Fig. 2(a)] were measured to confirm a higher compression ratio compared to conventional symmetric phase gratings and to find propagation distances with the best visibility for each TAI. One measured intensity modulation is shown in the background of Fig. 2(b) for the TAI of 6.8 µm period at 15 keV. Some images from the Talbot carpet scans are provided in Supplement 1 and compared with theoretical simulations. Similar scans have also been performed at 10 keV and 20 keV with the respective TAIs, and some key parameters are listed in Supplement 1.

For comparison, a speckle pattern generated by a sheet of P1000 sandpaper representing a random phase modulator was also measured analogously to the Talbot carpet scans. The achieved visibility and its standard deviation according to Eq. (1) is plotted with increasing propagation distance in Fig. 2(c) for all scans at 15 keV beam energy. To compare with recent literature demonstrating periodic x-ray DOEs discussed earlier, we plotted the peak performance of the different TAIs in Fig. 2(d). We only included research performed with single 2D gratings and modulators operated at synchrotron facilities for an appropriate comparison. The theoretical limit imposed by the PSF was calculated by convolving a periodic 2D square array of intensity points (resembling an ideally focusing modulator) with a Gaussian of σ=1.5µm estimating the blur of the used detector.

**Fig. 3.**
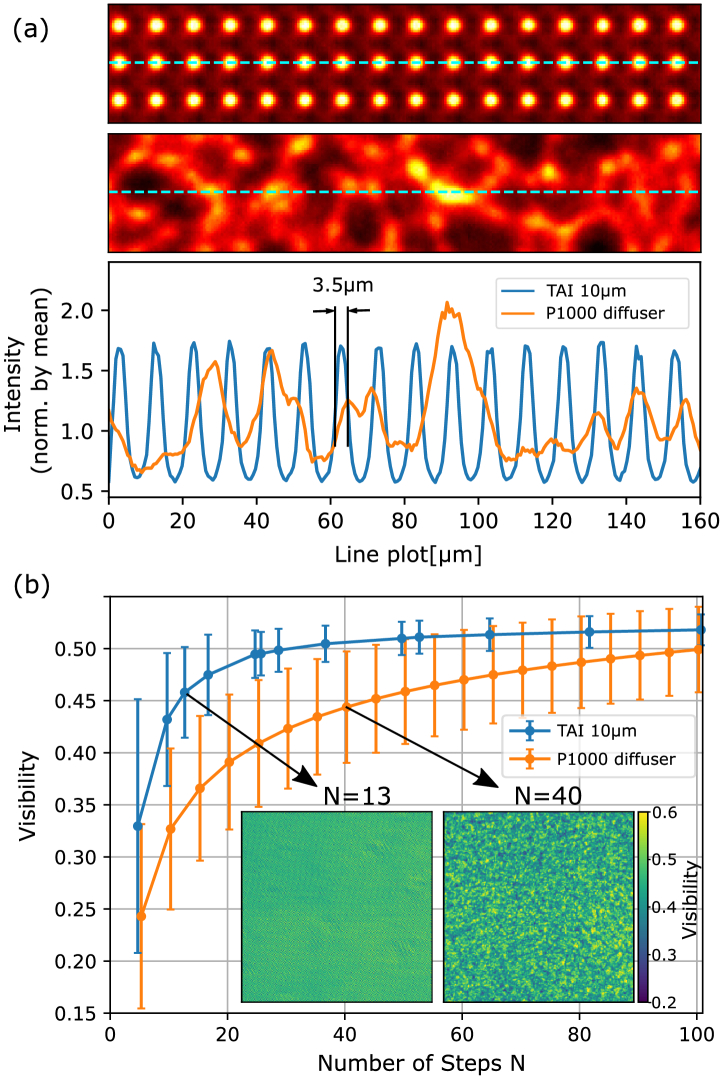
(a) Comparison of intensity modulations created by the 10 µm TAI and the P1000 diffuser with respective line plots illustrating the speckle sizes and densities. The images are normalized to the respective mean of the overall intensity values for a better comparison. (b) Comparison of visibilities and their standard deviations (error bars) with different numbers of steps for the TAI and the P1000 diffuser, including visibility maps for the TAI at N=13 and the P1000 at N=40 .

For a better spatial resolution, a 1D stepping acquisition of the TAI for bi-directional phase sensitivity was evaluated and compared to a stepping procedure with the P1000 diffuser. The scheme, similarly applied before with absorption grids [11], is illustrated in Fig. 2(b). The stepping direction and range is chosen along a vector consisting of multiple unit cell vectors of the grating structure (e.g., a=1 and b=3 ) such that every unit cell is sampled uniformly by the periodic intensity maxima. This can be achieved by rotating the grating in an angle of arctan(a/b) to the stepping direction and performing $N=a^{2}+b^{2}=10$ steps. Such a homogeneous sampling can be achieved for different integers a and b when they are coprime. It is noteworthy that homogeneous sampling does not necessarily have to be a quadratic lattice and can also be achieved with other rotation angles relative to the pixel matrix when the stepping range and step size can be precisely controlled. This approach, however, assumes that all foci sampling the unit cell have a very similar shape, which can be compromised by fabrication-related deficiencies. Figure 3(a) shows a comparison of the intensity pattern generated by the 10 µm 2D TAI and the P1000 diffuser together with two line plots illustrating differences in the speckle densities and sizes. Stronger spots appear occasionally in the speckle pattern and would result in higher visibilities compared to the TAI when large pixel windows would be used for analysis according to Eq. (1). For a realistic comparison close to the mode of imaging operation, stepping sets with different numbers of steps N were composed from measured data, and the overall visibility and its standard deviation were evaluated in every pixel. In the case of the TAI, the 1D stepping scheme discussed above was used, and for the P1000 diffuser, a spiral stepping with an inter-step distance larger than the average speckle size was used to emulate a random stepping without repeated or very similar steps. The mean of the visibility and its standard deviation depending on the number of steps are shown in Fig. 3(b). Exemplary visibility maps for the TAI with N=13 and P1000 with N=40 steps are plotted for comparison.

**Fig. 4.**
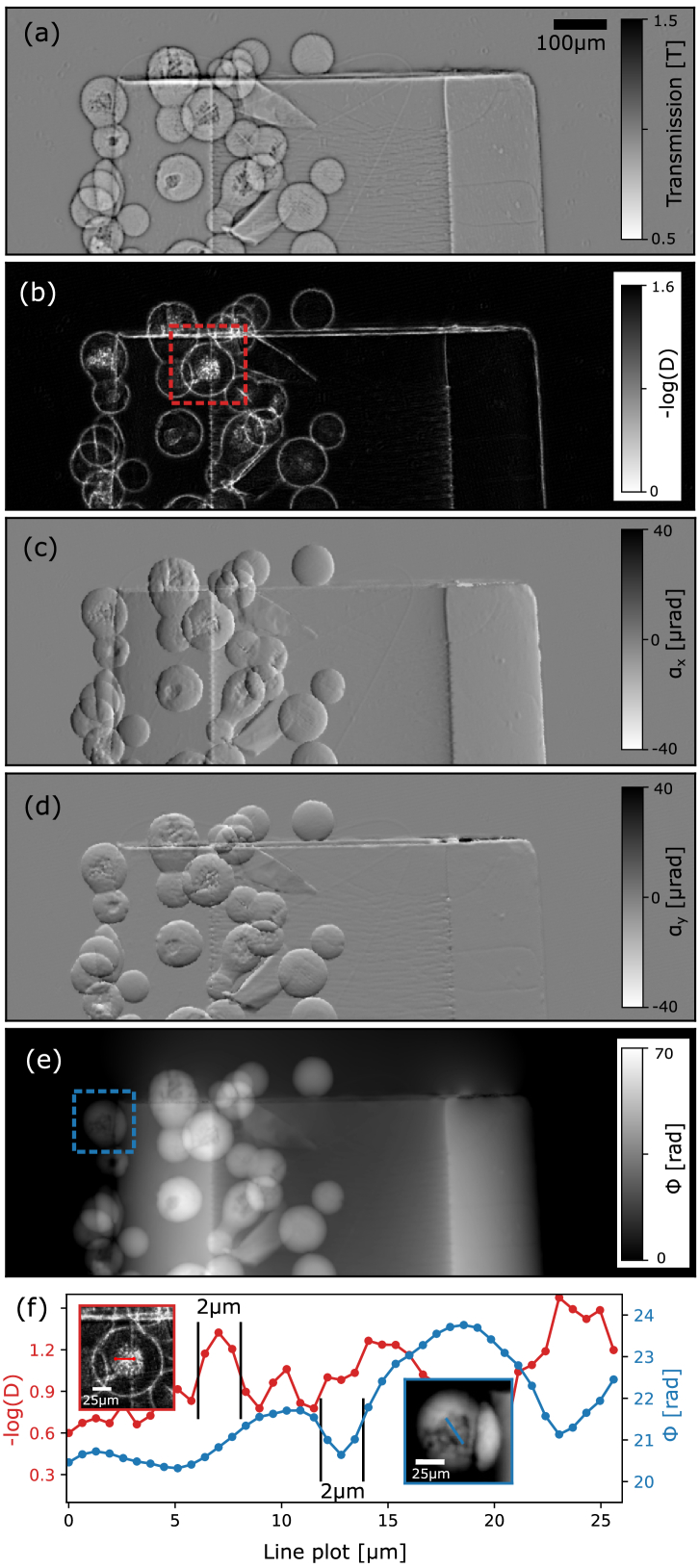
(a) Transmission image, (b) dark-field image, differential phase contrast image in (c) x direction and (d) y direction as processed by UMPA and (e) the integrated phase image. (f) Line plots of the dark-field and phase signal from some selected features shown in the red and blue ROIs, respectively.

To demonstrate the imaging capabilities of the system, a sample consisting of silica particles with partially porous inner structures glued to a plastic micropipette tip was used. It was measured with the 6.8 µm TAI placed 170 mm from the detector at 15 keV beam energy. The sample was at d=70mm propagation distance to the detector, and the TAI was stepped linearly according to the scheme discussed above. The acquired data was processed using unified modulated pattern analysis (UMPA) [14], which is a flexible and robust algorithm suitable for both SBI as well as phase retrieval with periodic modulation patterns. More details about the processing and phase integration are provided in Supplement 1. Figures 4(a)–4(d) show different image channels acquired with N=17 steps and processed with a window size of 5 pixels (0.64 µm effective pixel size). Some line plots in Fig. 4(f) show selected small features of the silica particle from the dark-field and the phase channel together with inlets, which were acquired with N=25 steps and processed with a window size of 3 pixels.

**Fig. 5.**
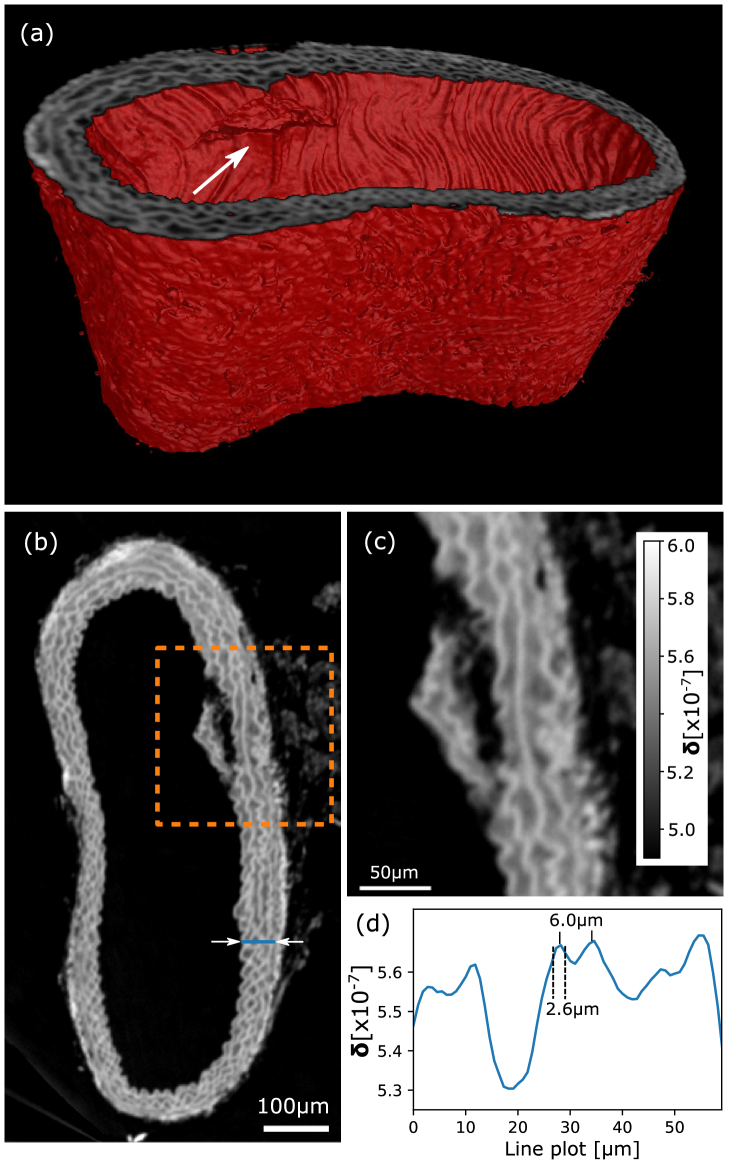
(a) Rendering of a mouse aorta CT phase scan depicting a fissure in the vascular wall (white arrow) and (b) a respective slice showing its lamellar structure. (c) Magnified image of the ROI in (b). (d) Line plot [see blue line in (b)] showing that two lamellae distanced 6 µm apart can be well resolved.

An entire CT scan of a paraffin-embedded mouse artery (brachiocephalic) taken from an “old” mouse (20 months) with atherosclerosis was performed at 20 keV acquiring N=15 steps per projection. Since a larger FoV was required to encompass the sample, the detector configuration was changed to a 5× optical magnification with an effective pixel size of 0.91 µm. The 6.8 µm TAI was placed 170 mm upstream the detector, and the sample was mounted at a propagation distance d=150mm . In total, 4001 projections were acquired over 180° sample rotation. A specially adapted matching algorithm (described in detail in Supplement 1) was developed to find the most suitable flat field for every sample frame. The reconstruction was performed by filtered back-projection of the integrated phase images using a Ram-Lak filter. Figure 5(a) shows a 3D rendering of a section of the artery, and an arrow is depicting a fissure in the vascular wall. The latter is shown in the respective slice [Fig. 5(b)] with a magnified view in [Fig. 5(c)]. A line plot through the lamellar structure [see blue line in Fig. 5(b)] is plotted in Fig. 5(d) to estimate the achieved resolution. Further quantitative analysis related to the resolution is included in Supplement 1.

## 4. RESULTS AND DISCUSSION

### A. Visibility-Distance Analysis

As predicted by the simulation, the evaluated TAIs create strongly modulated patterns near the fractional Talbot distances. In the case of the 5 µm TAI, the first visibility peak [see Fig. 2(c)] appears in the vicinity of 100 mm and the second around 400 mm, which corresponds to $d_{T}/6$ and $2d_{T}/3$ . Note that the highest visibility does not have to be necessary on the exact position of the fractional Talbot distance, since strong focusing occurs even before and after $d_{T}/6$ as the simulations show. The 5 µm TAI can be used at $2d_{T}/3$ for high-sensitivity measurements, as it delivers comparable visibility to the 6.8 µm TAI at a propagation distance of 420 mm. Due to its smaller period, the 5 µm TAI allows a finer sampling with an equal number of phase steps compared to the 6.8 µm TAI. The latter achieves higher visibility at $d_{T}/6$ , which is to be expected due to the PSF blur. The highest visibility of the TAIs for 15 keV is achieved by the 10 µm TAI at 380 mm, which is close to the $d_{T}/6$ modulation. The speckle visibility of P1000 sandpaper increases constantly with propagation distance; however, it is still always below the 10 µm TAI and suffers by a much higher standard deviation. Even at higher propagation distances beyond 500 mm, the speckle visibility increases slowly and reaches 0.42 at 1000 mm. In that range, the limited beam coherence additionally degrades the contrast. This limitation is even more severe for laboratory-based sources with larger focal spots and divergent beams. With longer propagation distances, the geometrical source blur decreases the contrast, and the intensity drops by the inverse-square law. Hence, the TAIs show pivotal advantages for creating high contrast modulations at small periods and shorter propagation distances. As Fig. 2(d) shows, the evaluated modulators are much closer to the theoretical limit in terms of the period-to-visibility ratio compared to other recent examples. Compared to [16], the 6.8 µm TAI achieves similar visibility at about 10× smaller periods and enables, therefore, e.g., single-shot imaging at about 1 order of magnitude better resolution. Compared to, e.g., [12], the configuration of the 5 µm TAI gives a 6× higher visibility, which allows us to shorten the measurement time and lower the dose significantly. Compared to the refractive lens arrays [17] creating 20×20 foci with 50 µm period and superior visibility, the 6.8 µm TAI creates about 1000×360 foci across the FoV enabling both a higher resolution and a larger FoV in single-shot imaging mode. In terms of the visibility-to-period ratio [indicated by the dashed lines in Fig. 2(d)], the TAIs are more than a factor of 2 better and, therefore, also allow a more dose-efficient sampling compared to, e.g., [17], as further discussed in Supplement 1.

### B. Comparison of TAI with P1000 Diffuser

A comparison of periodic phase modulators with diffusers is not straightforward as they do not have a distinct period and the reached visibility either depends on the analysis window size w or the number of steps when a pixel-by-pixel approach is used. Furthermore, it depends on the beam energy, coherence, and propagation distance. Still, a realistic evaluation of the achieved visibility in a phase stepping process with increasing N shown in Fig. 3(b) emphasizes the benefits of the TAI. The visibility, as well as its standard deviation, saturate after N=20 for the TAI, while the P1000 diffuser requires much more steps to achieve comparable results and its standard deviation decreases only slowly with increasing N . With N=13 , an overall higher visibility with a lower standard deviation is achieved with the TAI than for the P1000 diffuser with N=40 steps. A detailed evaluation considering the intensity gradients of the modulation pattern, which is the key factor for a good phase sensitivity [35], is given in Supplement 1. Using the TAI instead of the P1000 diffuser, an improvement by a factor of 6 in dose efficiency is estimated. It is attributed to strong intensity gradients and their high density, as well as their periodic nature, which allows a highly efficient sampling.

### C. Projectional Imaging

As shown in Fig. 4, almost artifact-free images with a high resolution can be acquired with a relatively low number of steps. In the transmission image [Fig. 4(a)], features close to edges or grainy regions are distorted by edge enhancement effects (halos around spherical shapes) due to a long propagation distance to the detector. The dark-field image [Fig. 4(b)] shows characteristic enhancement of edges, but also porous structures inside the spheres are well recognizable. Some faint periodic artifacts are present in the dark-field image probably caused by insufficient sampling ( N=17 steps). They can be avoided by choosing a larger window size in UMPA processing; however, this will also reduce the resolution. Both differential phase contrast images in the x [Fig. 4(c)] and y [Fig. 4(d)] direction show artificially rough edges of the silicon spheres, which are probably the result of incompatible phase sampling and window size. However, those artifacts are hardly recognizable in the integrated phase image [Fig. 4(e)]. The sensitivity of the differential phase contrast images in both directions is similar ( $\sigma_{x}=175\;\;nrad$ , $\sigma_{y}=186\;\;nrad$ ), confirming an overall good sampling in both directions. Line plots provided in Fig. 4(f) show that features of about 2 µm full width at half-maximum (FWHM) can be resolved in both the dark-field as well as the phase image. That is close to the theoretical limit of UMPA phase retrieval imposed by the window size [15] (in this case w=3 ) as well as the PSF of the detector.

### D. Computed Tomography

The phase CT scan of the unstained mouse artery demonstrates the potential for high-resolution, quantitative, non-destructive 3D virtual histology. The vessel was extracted from an aged animal with a pathological condition (atherosclerosis) and the slice in Fig. 5(b) shows in detail that there is a measurable fissure of the elastin fibers, commonly seen in frail, old animals and in humans. The contrast between the background (paraffin) and the bright elastin fibers is about $0.76\times 10^{-7}$ , and the background noise level in the paraffin matrix is $0.015\times 10^{-7}$ . Hence, a contrast-to-noise ratio (CNR) of >50 between these soft matter components is achieved. A line plot of the reconstructed refractive index decrement δ values in Fig. 5(d) shows that two different lamellae distanced about 6 µm from each other can still be well resolved and neighboring features with an FWHM of about 2.6 µm are well distinguishable. Well-defined edges quantifying the resolution at paraffin-tissue interfaces evaluated further in Supplement 1 also suggest a resolution of about 3 µm achieved with N=15 phase steps per projection. A recent comparable work using state-of-the-art SBI achieved about 8 µm resolution at N=20 phase steps at an energy of 26.3 keV [36]. Thus, we are approaching the limit imposed by the detector PSF and come close to the resolution of propagation-based phase imaging. The latter is unprecedented in resolution among non-ptychographic full-field techniques; however, it is less sensitive to small density differences since it is based on the Laplacian of the phase. Furthermore, the most commonly used reconstruction algorithm [37] requires various assumptions about the sample, resulting in limited applications and difficulties for quantification.

## 5. CONCLUSION

We designed and evaluated 2D TAIs with small periods for 10 to 20 keV x-ray beam energy. They create periodic foci with higher compression ratios and visibilities than conventional 2D phase gratings and have many advantages over absorption gratings or apertures, refractive micro-lens arrays, or random phase modulators used in SBI. The short periods also allow a finer and more efficient phase sampling for higher resolution and sensitivity. In this work, we addressed the drawbacks of grating-based imaging (GBI) compared to SBI, which are, e.g., listed in [38]. We avoided using absorptive elements and employed only one, thin phase modulator reducing the setup complexity. Furthermore, we reached bi-directional sensitivity with 1D linear phase stepping and achieved unprecedented resolution with a low number of phase steps. For 1D stepping, an angular alignment of the TAI remains necessary and depends on the number of steps N . Using a 2D stepping stage, the alignment becomes obsolete, and the experimental setup is virtually identical to that of SBI. A disadvantage of periodic modulators (such as the TAIs) is the limited dynamic range for measuring phase-induced displacements of the intensity pattern. Similarly to GBI, this can result in phase wrapping, which is usually not the case with random modulators, where a displacement larger than the speckle size can be matched. Furthermore, TAIs operate best at designed propagation distances, which strongly depend on the period, while random diffusers provide a broader range with relatively high visibility. Strong and sharp spatial modulations of δ (e.g., at edges, air bubbles) remain a problem, since they deteriorate the intensity pattern to a degree that it cannot be reasonably matched with the reference pattern. This could be addressed by tuning the sensitivity (sample-detector distance, beam energy, larger modulator period) or excluding the concerned pixels from the CT reconstruction using, e.g., advanced iterative CT algorithms [39]. Another possibility would be to apply ptychographic phase retrieval algorithms, which have been shown beneficial for resolution with structured illumination [40]. Future developments will include further optimization of the CT acquisition schemes to reduce the measurement time, radiation dose, and setup stability issues. In particular, a fly-scan CT [41] with continuous sample rotation and frame rate at every phase step may improve the current protocol. Although a 1D stepping was successfully employed, a 2D stepping will simplify alignment and provide more flexibility for alternative sampling schemes. A detailed quantitative analysis of the absolute δ values, as well as comparison to propagation-based phase tomography, is ongoing and will be addressed in future work.

The TAIs can be designed to operate efficiently in laboratory-based x-ray imaging systems with lower source coherence, shorter propagation distances, lower detector resolution, and higher x-ray energies. When SBI is performed with higher energies, strongly absorbing diffusers (e.g., steel wool [42]) are used, or multiple layers of sandpaper have to be stacked (e.g., up to 20 layers for 65 keV [13]) to achieve decent speckle visibility. Periodic modulators like the TAIs proposed in this work can be designed for significantly higher energies on thin substrates. Current anisotropic silicon etching technology achieves aspect ratios beyond 1:20, making modulators with 10 µm period possible for 100 keV and above on 250-µm-thick silicon substrates.

Beyond high-resolution or single-shot dynamic phase imaging, the discussed TAIs can be also used in wavefront sensing, x-ray optics characterization, adjustment and focusing of scintillator screens in 3D, or for recently demonstrated full-field structured illumination super-resolution x-ray microscopy [19,43]. Using high power, laboratory-based x-ray sources with absorptive source gratings [5], the discussed TAIs may be also used in medical Talbot-Lau-based imaging systems to gain bi-directional sensitivity and increased visibility with shorter setups compared to conventional binary symmetric phase gratings.

## Data Availability

Data underlying the results presented in this paper may be obtained from the authors upon reasonable request.

## Supplemental document

See Supplement 1 for supporting content.
