# Supplementary material for: High-resolution and sensitivity bi-directional x-ray phase contrast imaging using 2D Talbot array illuminators
Source: Optica. 2021 Dec 10;8(12):1588–95. doi: 10.1364/OPTICA.441004 (PMC10567101; doi:10.1364/OPTICA.441004)
Supplement: Supplementary file 1 [file optica-8-12-1588-s001.pdf]

Supplemental Document

OPTICA

High-resolution and sensitivity bi-directional x-ray phase contrast imaging using 2D Talbot array illuminators: supplement

ALEX GUSTSCHIN,[1,†,*] MIRKO RIEDEL,[1,2,†] KIRSTEN TAPHORN,[1] CHRISTIAN PETRICH,[1] WOLFGANG GOTTWALD,[1] WOLFGANG NOICHL,[1] MADLEEN BUSSE,[1] SHEILA E. FRANCIS,[3] FELIX BECKMANN,[2] JÖRG U. HAMMEL,[2] JULIAN MOOSMANN,[2] PIERRE THIBAULT,[4] AND JULIA HERZEN[1]

[1]*Department of Physics and Munich School of Bioengineering, Technical University of Munich, 85748, Garching, Germany*
[2]*Institute of Materials Physics, Helmholtz-Zentrum Hereon, Max-Planck-Str. 1, 21502 Geesthacht, Germany*
[3]*Department of Infection, Immunity and Cardiovascular Disease, Medical School, University of Sheffield S10 2RX, UK*
[4]*Department of Physics, University of Trieste, Trieste 34217, Italy*
[†]*These authors contributed equally to this work.*
[*]*Corresponding author:* alex.gustschin@ph.tum.de

This supplement published with Optica Publishing Group on 10 December 2021 by The Authors under the terms of the Creative Commons Attribution 4.0 License in the format provided by the authors and unedited. Further distribution of this work must maintain attribution to the author(s) and the published article's title, journal citation, and DOI.

Supplement DOI: https://doi.org/10.6084/m9.figshare.16918225

Parent Article DOI: https://doi.org/10.1364/OPTICA.441004

High resolution and sensitivity bi-directional x-ray phase contrast imaging using 2D Talbot array illuminators: supplemental document

The supplementary document presents further details on the experimental methods and data processing. We address simulation, design, fabrication, and evaluation of the TAIs. Further, we give a brief characterization of the P1000 diffuser used for comparison with the TAIs and describe the scheme which was employed for the respective visibility analysis. After that, we elaborate on the imaging and tomography experiments, image processing, and computed tomography (CT) reconstruction. We give some details on the angular sensitivities reached experimentally with different numbers of steps for the 6.8 µm TAI using the proposed 1D stepping approach. Finally, we describe two methods to estimate the spatial resolution of the CT scan of the murine artery.

1. DETAILED MATERIALS AND METHODS

A. Simulation, Design, and Fabrication of TAIs

The simulation of coherent wave propagation was performed using the Fresnel diffraction formula,

$$\Phi(x,y,z) = \exp(ikz)F^{-1}\left[F(\Phi_0)\exp\left(-i\frac{k_x^2+k_y^2}{2k}z\right)\right], \tag{S1}$$

where $\vec{k}$ is the wave vector, $k = |\vec{k}|$ its modulus, F denotes the Fourier transform (F^{-1} is the inverse) and $\Phi_0 = \Phi(x,y,z=0)$ is the coherent wavefront created by the grating at $z = 0$. The intensity I measured by a detector is calculated by $I = |\Phi(x,y,z)|^2$ and is denoted as Fresnel image following the terminology from [1]. Cross-sections of I along the propagation direction are denoted as Talbot carpets. The calculations were performed with an area of 20×20 periods at 100 nm resolution. We assumed the TAIs as pure phase objects as the attenuation by the modulator height is negligible at the respective beam energies. The Talbot carpet of the central 3 periods is shown in Figure S1 (a). To obtain realistic visibility values the point spread function (PSF) of the detector had to be taken into account. For that, we convolved the simulated Fresnel images with a Gaussian kernel of $\sigma = 0.7$ µm in Figure S1 (b) for a better comparison with the measured data (c). The design of the employed 2D phase modulators for x-ray applications was driven by multiple factors. They have to be (1) adapted to a good trade-off between visibility and period with a given detector PSF, (2) flux efficient using an x-ray transparent material, (3) resistant to high doses of radiation, and (4) easy to fabricate using current microprocessing technologies. Silicon was found to be a good choice given its widespread use in the semiconductor industry. The TAIs were fabricated according to our design specifications by 5microns GmbH (Illmenau, Germany) via UV lithography and deep reactive ion etching on 250 µm thin silicon wafers. Multiple arrays of 10 mm×10 mm size were patterned and etched to different depths, which depend on the desired phase shifts at the respective beam energies. Table S1 gives an overview of the different TAIs including the design parameters and the measured values for the height and the duty cycle based on scanning electron microscopy (SEM) images of the profiles. Some images of the gratings and micrographs of the profiles are shown in Figure S2.

It is noteworthy that such modulators can be easily fabricated for significantly smaller periods than evaluated in this work. A similar structure can be patterned with 2 µm period with e.g. electron beam lithography and etched with an aspect ratio of 1:10 with DRIE reaching similar heights as the modulators for 15 keV. In our case, however, periods smaller than 5 µm would not provide reasonable performance with the used detector. Furthermore, TAIs for significantly higher energies than evaluated in this work can be fabricated. At e.g. 10 µm period such modulator can be fabricated for energies >100 keV with aspect ratios routinely realized by conventional DRIE.

Table S1. **Fabrication-related parameters of the evaluated TAIs.**

Modulator	Period[μm]	Energy[keV]	Height [μm] (design/actual)	Duty Cycle (design/actual)
TAI 5-10	5.0	10	8.5 / 8.1	0.33 / 0.31
TAI 10-10	10.0	10	8.5 / 9.1	0.33 / 0.31
TAI 5-15	5.0	15	12.8 / 12.5	0.33 / 0.30
TAI 6-15	6.8	15	12.8 / 13.5	0.33 / 0.29
TAI 10-15	10.0	15	12.8 / 14.9	0.33 / 0.31
TAI 5-20	5.0	20	17.1 / 15.5	0.33 / 0.27
TAI 6-20	6.8	20	17.1 / 17.1	0.33 / 0.29
TAI 10-20	10.0	20	17.1 / 18.7	0.33 / 0.30
TAI 13-20	13.6	20	17.1 / 19.9	0.33 / 0.31

Table S2. **Experimental parameters for the evaluated phase modulators. *For the P1000 diffuser the average speckle size is given instead of the FWHM focus size.**

Modulator	Energy[keV]	Analysis window [px]	FWHM Foci [μm]	Maximum Visibility
TAI 5-10	10	8	1.9	0.27
TAI 10-10	10	16	4.2	0.58
TAI 5-15	15	8	2.0	0.31
TAI 6-15	15	12	2.5	0.42
TAI 10-15	15	16	3.5	0.54
P 1000	15	18	6.9*	0.36
TAI 5-20	20	8	2.4	0.29
TAI 6-20	20	12	2.8	0.45
TAI 10-20	20	16	3.9	0.61
TAI 13-20	20	22	5.3	0.68

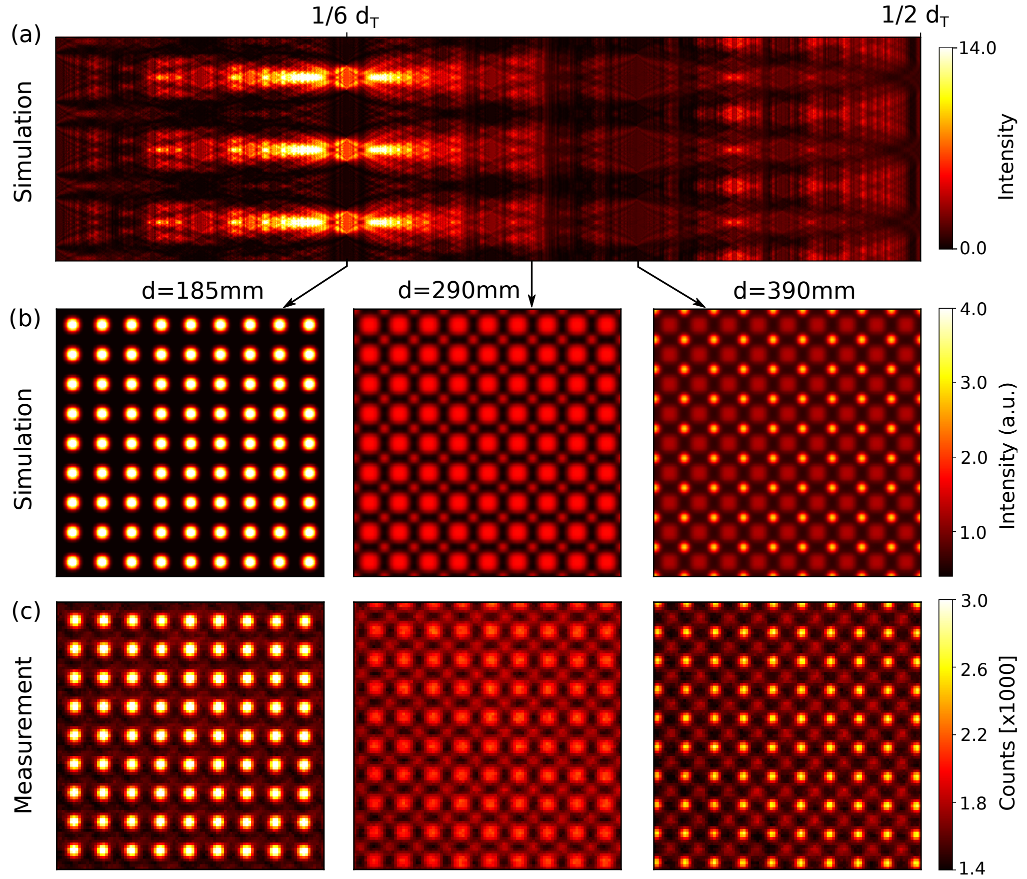

Fig. S1. (a) Simulation of the Talbot carpet for the TAI 6-15 at 15 keV (see Table 1) and (b) some Fresnel images at selected propagation distances after a convolution considering the detector PSF. (c) Acquired Fresnel images at the same propagation distances as in (b) agree well with the simulated data.

B. Measurement of Talbot Carpets

Multiple Talbot carpet scans were performed at the micro-tomography end-station of the P05 imaging beamline (IBL) at PETRA III at DESY. The radiation is generated by an undulator source with the dimensions of 36 µm × 6 µm about 86 m before its exit point to the experimental chamber and is monochromatized by a double crystal monochromator yielding a bandwidth of approximately $\Delta E/E = 10^{-4}$ [2]. The detector was a 50 µm thick lutetium-aluminium garnet (LuAG) scintillator optically coupled (10× magnification) to a CMOS camera (CMOSIS CMV20000 chip) resulting in an effective pixel size of 0.64 µm[3]. Images were taken every 5 mm from 40 mm to 540 mm distance to the detection plane with an exposure time of 200 ms. All measured TAIs with the respective periods, duty cycles, and design energies are given in Table S1. The maximal visibilities according to equation S3 and typical FWHMs of the foci created by the TAIs are listed in Table S2. To explore if the different beam coherence in x- and y-direction has an influence on the intensity modulation we analyzed 1000 randomly selected foci of the 5 µm TAI at 15 keV and 420 mm propagation distance. We extracted the FWHM in x- and y-direction from each 2D Gaussian fit and obtained mean values of $\text{FWHM}_x = 2.033 \pm 0.111$ µm and $\text{FWHM}_y = 2.004 \pm 0.108$ µm. Hence, the difference in horizontal and vertical source size did not affect the visibility and resolution at the discussed propagation distances. Note, that in case of the 5 µm TAI where both highly modulated Talbot orders are visible around 100 mm ($d_T/6$) and 400 mm ($2d_T/3$) propagation distance (see Fig. 2(c) in the main work) only a slight visibility drop by 0.015 (≈5%) is visible. This is also an indication that limited beam coherence hardly degrades the contrast. For laboratory sources with polychromatic spectra and more extended source sizes a stronger visibility drop in a higher Talbot order would be observable, as they have a lower spectral

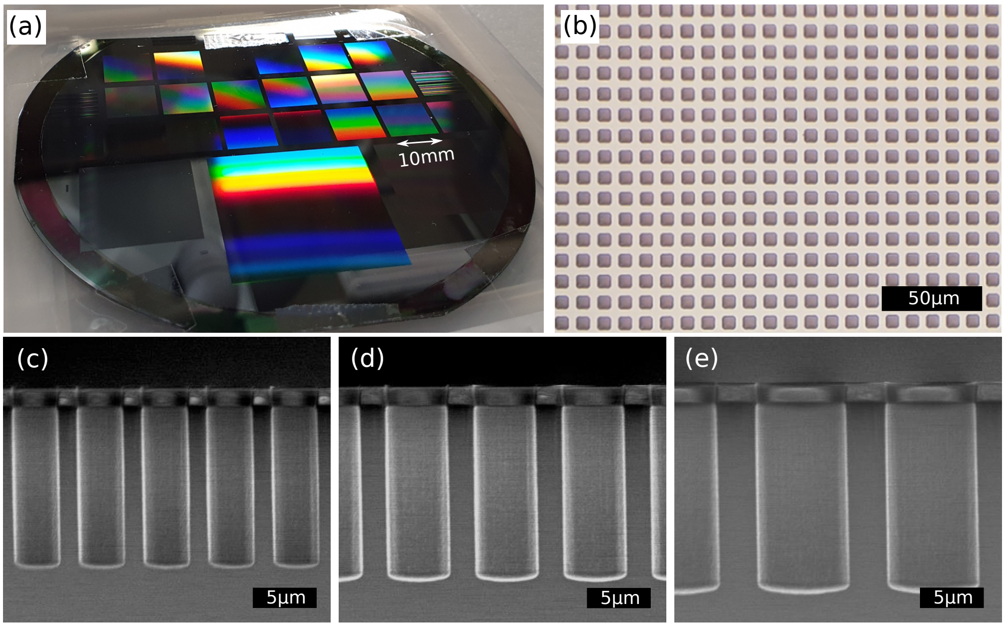

Fig. S2. (a) Multiple Talbot Array Illuminators (TAIs) with different periods on a 100 mm silicon wafer. (b) Light microscopy image of the 10 µm array. (c-e) SEM profile micrographs of the 5 µm array (c), 6.8 µm array (d) and 10 µm array (e) etched simultaneously on the same wafer. Images (c-e) were kindly provided by 5microns GmbH (Illmenau, Germany).

acceptance [4, 5]. Figure S1(c) shows exemplary ROIs of the measured Fresnel images in different propagation distances acquired with the TAI 6-15 at 15 keV. The patterns match the simulated data (S1(b)) well, except that their contrast is somewhat lower due to the detector PSF.

C. Characterization of the P1000 Diffuser

A sheet of P1000 sandpaper (representative for a diffuser used in SBI) was measured with the same protocol to study and compare its speckle contrast with the evaluated TAIs. Figure S3(a) shows a 1000 × 1000 pixel ROI from the center of the speckle pattern at 15 keV and a propagation distance of 380 mm. An azimuthally averaged and normalized plot of the 2D autocorrelation function of the speckle pattern is given in Figure S3(b). The half-width is at about 5.4 pixels resulting in a full width at half maximum (FWHM) of about 6.9 µm which is a good measure of the average speckle size. The minimum at about 18 pixels (11.5 µm) is an average distance at which a maximal intensity modulation is expectable and was used as window size in the visibility determination. The 2D autocorrelation is also given as an inlet in Figure S3(b) showing that the beam coherence in both directions was high enough to not impair the speckle contrast at that propagation distance. Speckle patterns are characterized by different visibility conventions which are usually not comparable to each other [6]. Figure 3(c) shows the visibility map determined in a window of 50 × 50 pixels by standard deviation σ_w:

$$V_{std} = \frac{\sigma_w}{\overline{I_w}}, \tag{S2}$$

where $\overline{I_w}$ is the mean of the intensity values in the window. Figure S3(d) shows a visibility map determined by the minimal (I_{min}) and maximal (I_{max}) intensity pixel values in a window size of 18 pixels by

$$V = \frac{I_{\text{max}} - I_{\text{min}}}{I_{\text{max}} + I_{\text{min}}}. \tag{S3}$$

This convention is also used for the comparison with the TAIs at respective window sizes. Figure S3(e) shows a visibility map extracted pixel-by-pixel by formula S3 from a stepping with $N = 40$ random steps. The averages and standard deviations of the respective visibility arrays are

given in the right corners of the maps respectively. Note that Figure S3(c) has a different leveling of the colormap.

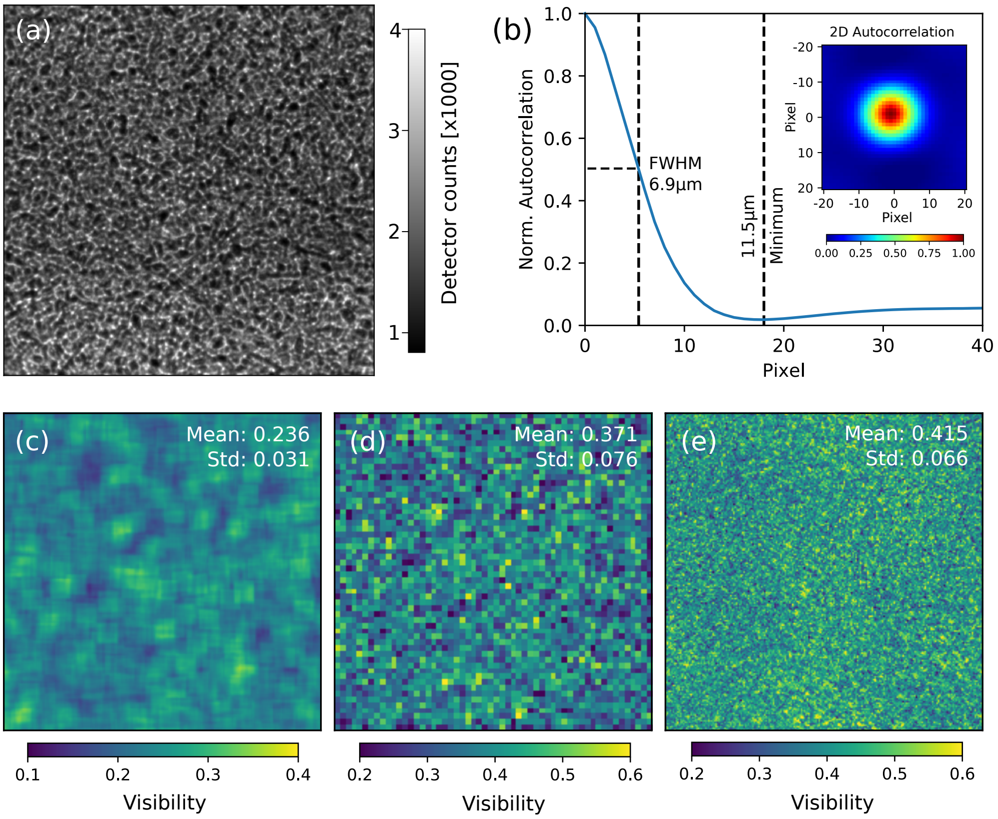

Fig. S3. (a) Measured speckle pattern (1000 × 1000 pixel central ROI) at 380 mm propagation distance. (b) Azimuthally-averaged plot of the 2D autocorrelation (seen in the inlet) of the speckle pattern suggesting an average speckle size of 6.9 µm. (c) Visibility map calculated by convention from equation S2 with a window size of 50 × 50 pixels and (d) calculated by equation S3. (e) Visibility map calculated pixel-by-pixel from a spiral stepping with $N = 40$.

D. Linear 1D phase stepping with bi-directional sensitivity

For a robust and high-resolution phase retrieval every pixel should be modulated with high contrast in both directions during phase stepping. The intensity maxima created by the TAI can be understood as sampling points which should be homogeneously distributed over the pixel matrix. This can be easily achieved by mounting the TAI on a 2D linear positioning stage. With random phase modulators used e.g. in SBI bi-directional sensitivity can be also achieved with linear 1D stepping as the random speckle pattern contains intensity gradients in all directions [6]. In this work, we use a phase stepping scheme with periodic 2D modulators that allow achieving a homogeneous sampling and therefore bi-directional phase sensitivity with a 1D linear phase stepping of the modulator. The principle is illustrated in Figure S4 (a) and (b). The points on a square grid with the same color represent the foci created by the modulator and different colors represent different stepping positions. The stepping direction and range depicted by the dashed arrow are chosen as a linear combination of several unit cell vectors of the periodic grating structure. Here a and b are the catheti of the triangle given in the unit of the period. When stepping over the diagonal in an integer number of steps N and a step distance p' the Pythagorean theorem in the rectangular triangle yields:

$$a^2 + b^2 = (Np')^2. \tag{S4}$$

For a homogeneous square sampling the step distance p' is also the sampling period and has so conform to $Np'^2 = 1$ assuming that the period of the modulator is 1. Combining this

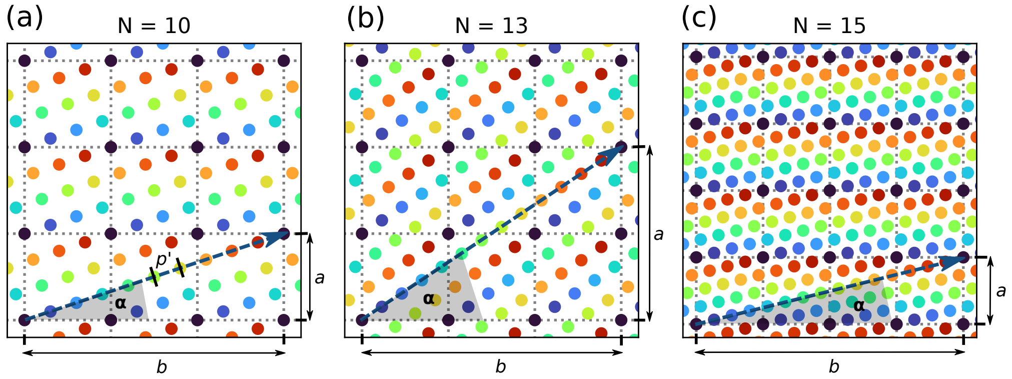

Fig. S4. Illustration of different phase stepping schemes. Points of the same color represent the periodic intensity spots created by the TAI in a square lattice. Different colors illustrate different stepping positions and the dashed arrows depict the stepping direction and range. The stepping direction is tilted in an angle α relative to the grid of the TAI. Exemplary stepping schemes ($a = 1, b = 3$) with $N = 10$ steps (a) and ($a = 2, b = 3$) with $N = 13$ steps (b). Linear stepping with periodic modulators also allows other schemes such as e.g. an irregular hexagonal sampling shown in (c) with $a = 1, b = 4$ and $N = 15$ steps.

with equation S4 yields $N = a^2 + b^2$ for the number of steps. Note here that a and b have to be coprime, otherwise, some steps will produce redundant sampling points. The sampling grid is orientated in an angle $\alpha = \arctan(a/b)$ to one direction of the modulator grid. Hence, mounting the modulator in an angle α between the stepping direction and the modulator lattice a sampling with equal sensitivity in both directions can be achieved in theory. In practice, the differences in sensitivity in both directions due to unprecise stepping and alignment angle deviations were relatively small as seen in Table S3. The image quality mainly depends on the sampling period p' as both the resolution and the sensitivity increase with a better sampling. Since $Np'^2 = 1$ the sampling can be adjusted by choosing a suitable N that satisfies equation S4. Figure S4 (a) shows an example with $a = 1, b = 3$ with $N = 10$ and (b) with $a = 2, b = 3$ with $N = 13$ steps. Exploiting the periodicity of the modulator also other possible sampling schemes can be achieved. In Figure S4 (c) a stepping with $a = 1, b = 4$ with $N = 15$ steps results in an irregular hexagonal sampling, which has a higher sampling efficiency than square sampling. Note that in this example the hexagonal grid is not regular, i.e. the hexagons are slightly compressed in one direction. While using this principle can reduce the setup complexity as it requires only one stepping direction the modulator angle to the linear axis has to be carefully adjusted for every stepping scheme.

E. Visibility Comparison of 10µm TAI and P1000 Diffuser

All measured Fresnel images from the Talbot carpet scans were corrected with dark frames for read-out noise and strong pixel outliers (defective pixels) were replaced by its neighbors using a selective median filter. The visibility was extracted according to equation S3 from the pixel intensity values in a window size (see Table S2) similar to the period of each TAI. For the P1000 diffuser, the window size was chosen according to the first minimum of the azimuthally averaged autocorrelation function as described above. To evaluate the standard deviation of the visibility this analysis was performed in a 1000×1000 pixel array. The average visibility and its standard deviation represented by the error bars (only every 4th error bar was plotted for clarity) for the 15 keV measurements are plotted in Figure 2(c) of the main article.

To compare the realistic performance of the proposed 1D stepping scheme with a random stepping of the P1000 modulator, we composed stepping sets with different numbers of steps and calculated the resulting pixel-by-pixel visibility according to equation S3. Note that we chose equal propagation distances for the comparison of both modulators because different distances would result in incomparable flux at the detector and source coherence requirements when using a laboratory-based cone-beam system. If this aspect is irrelevant, a longer propagation distance could be used for the speckle pattern to reach a higher visibility. However, even at a measured propagation distance of 1000 mm the speckle visibility of P1000 was 0.42, which is significantly lower than that of the TAI at 380 mm (0.54).

Since a precise 2D stepping stage for the phase modulators was not available during the

experiments we used the following procedure to assemble sets of steppings with different N. For both the P1000 diffuser and the TAI we acquired the intensity modulation in a propagation distance of 380 mm (highest visibility with the TAI). In UMPA processing an efficient phase stepping consists of random diffuser positions with a step size significantly larger than the typical speckle size [6]. Despite a random selection, the stepping should not contain redundant or very similar frames (i.e. all diffuser positions should be more away from each other than the average speckle size). For that, we digitally shifted the P1000 pattern with sub-pixel interpolation on a spiral trajectory with an inter-step distance higher than the average speckle size and composed the shifted frames to stepping sets with a different number of frames N.

For the 2D TAI, the measured frames were rotated to an angle $\alpha = \arctan(a/b)$ and shifted digitally with sub-pixel interpolation to the respective positions, covering the range $a^2 + b^2$ in N steps according to the scheme described in the main article. For all stepping sets, the visibility was calculated pixel by pixel and its average and standard deviation were plotted versus the number of steps in Figure 3(c) of the main work. It should be noted that the sandpaper used for our comparison is not optimized for that energy and should not be considered as the ideal case of SBI. In general, a rigorous comparison between random and periodic phase modulators is difficult, since it requires finding the best random modulator for the set of parameters that a periodic modulator (e.g. TAI, grating, refractive lens array) is designed for. In our case, the P1000 sandpaper achieves relatively high visibility and is comparable to recent SBI literature. It should also be considered that a direct comparison in the imaging domain with the discussed modulators is difficult, since the created modulations are different in size. While the average speckle size is 6.9 µm the foci of the used TAIs are significantly smaller (e.g. 2.5 µm). Consequently, both data sets should be processed with different window sizes, which directly influence the resolution and sensitivity.

F. Imaging of Silica Particles

A sample consisting of porous silica particles glued on a plastic micropipette tip was used to test the resolution in both the phase and the dark-field modality. The 6.8 µm TAI was operated at a distance of 170 mm to the detector (same as for the Talbot carpet scans). The sample-detector distance was 70 mm. The TAI was slightly tilted to the detector pixel matrix and for different numbers of steps, the stepping distance was chosen accordingly to achieve a homogeneous sampling. To find optimal stepping parameters with different numbers of phase steps the stepping range was slightly varied. The best sampling is achieved when the sum of all phase steps is an array with a low standard deviation. This way we found good configurations for 13, 15, 17, and 25 phase steps (see Table S3) for the same TAI alignment angle. Additionally, multiple sets of flat fields were collected with the same scheme. Due to some possible experimental deficiencies like drifts of the beam, sample stage, or phase modulator mount as well as imprecise reproducibility of phase steps a digital matching of flat fields had to be performed prior to phase retrieval. For that, every sample frame was matched with the best acquired flat field using an image similarity algorithm on a sample-free area of the projection. To increase the precision the best flat field was further shifted digitally with sub-pixel interpolation in both directions to achieve optimal registration with the intensity pattern on the sample-free area. The phase retrieval was performed by Unified Modulated Pattern Analysis (UMPA) [7][1] with different numbers of phase steps and window sizes. Note that since the angle of the stepping direction to the grating pattern could not be controlled precisely, there are only a few configurations for N that produce a satisfactory sampling. Some of these configurations and the pixel-wise flat-field visibility and sensitivity are listed in Table S3. Both directional differential phase contrast images were used for a Fourier transform-based phase integration procedure described in detail in [8].

G. Specimen Preparation

A 17-month-old C57BL6/J mouse purchased from Charles River UK was transduced with AAV8-PCSK9 (6×10^{12} vg/mouse i.v.) and one week later was started on a high-fat Western type diet, (829100, Special Diet Services, UK) for 12 weeks to induce atherosclerosis. At 20 months of age, the mouse was terminally anesthetized with pentobarbital and perfusion fixed via the left ventricle, first with saline (2 ml) to remove the blood and then with 10% v/v buffered formalin (5 ml). Directly after this, the entire aortic tree (aortic arch, surrounding vessels, and heart) was dissected as a single entity and stored in fixation solution at 4 °C. The brachiocephalic artery was embedded in paraffin wax for the CT scan. All experiments were approved by the local review

[1] A Python implementation of UMPA can be accessed at https://github.com/pierrethibault/UMPA

board and the UK Home Office under license P5395C858. All mice were housed under standard light and dark conditions and had unlimited access to diet and water at all times.

H. X-ray phase CT of a Murine Artery

The murine artery embedded in paraffin wax features fine lamellar structures in the tunica media, well suitable for a good resolution and soft-tissue contrast benchmark. Since the sample was larger than the previous sample, the detector configuration had to be adapted for a larger FoV. The camera (Ximea CB500MG, 4.6 µm pixel size) was coupled to a 5× optical magnification lens system. A 100 µm Cadmium tungstate scintillator screen with a lower resolution but higher photon efficiency was focused at 20 keV yielding an effective pixel size of 0.91 µm. The FoV in this configuration was 7 mm × 2.5 mm. The sample was mounted on the tomography stage at a propagation distance of $d = 150$ mm to the detector. At every angle, 15 phase steps were performed with an exposure time of 200 ms each and a total of 4001 projections were acquired over 180° sample rotation. To avoid ring artifacts in the later reconstruction the sample was laterally shifted in-between projections. Sets of flat fields were acquired multiple times to account for setup-related drifts and instabilities. The total scan time (about 5h) was comparably long due to experimental overhead (mechanical stepping, sample shifts, sub-optimal frame acquisition parameters) and can be significantly reduced. The murine artery was embedded in a much larger paraffin block in a plastic sample vessel that required a much larger FoV in the horizontal direction than the sample itself. Hence, an optimized sample preparation would allow to reduce the width of the FoV and consequently the number of projections to satisfy the Nyquist sampling criterion. Furthermore, the acquisition time can be reduced by performing a whole sample rotation at every phase step instead of performing a stepping at every projection angle. Furthermore, time can be saved by performing a fly-scan with continuous sample rotation and detector readout [9]. Every frame was corrected for dark current, defective pixels, and flux fluctuation measured independently with the ring current of the synchrotron. To correct for grating drifts and imprecise positioning of the stepper flat fields were matched for every sample frame using the algorithm described previously for the silica particles. Bi-directional phase retrieval was performed by UMPA with a window size of 3 pixels. This step was most time-consuming requiring to process over 60000 frames and was therefore distributed on up to 20 servers of the DESY Maxwell cluster. Depending on the cluster load this required a few hours of computation time and is a further motivation to reduce the number of required phase steps with efficient modulators and sampling schemes. A subsequent Fourier transform-based phase integration [8] was used to retrieve the phase images. The CT reconstruction was performed via filtered back-projection (Ram-Lak filter) of the integrated phase images using the software X-Aid FDK Reconstruction Suite 2020.10.2 (Mitos GmbH, Garching, Germany). The reached sensitivity and visibility of the CT scan are given in Table S3.

2. PHASE SENSITIVITY, PERIOD AND VISIBILITY

The angular sensitivity of an imaging system refers to the ability of the setup to resolve small-angle deflections of the beam caused by refraction or scattering in the sample. The differential phase signal is directly related to the refraction angle α via [6]:

$$\begin{pmatrix} \partial\Phi/\partial x \\ \partial\Phi/\partial y \end{pmatrix} = \frac{2\pi}{\lambda} \begin{pmatrix} \alpha_x \\ \alpha_y \end{pmatrix} . \tag{S5}$$

To measure α the deflection of the intensity modulation must be resolved. Using the small-angle approximation it translates to

$$\begin{pmatrix} \alpha_x \\ \alpha_y \end{pmatrix} = \frac{p_{\mathrm{eff}}}{d} \begin{pmatrix} u_x \\ u_y \end{pmatrix} , \tag{S6}$$

where d is the distance from sample (placed after phase modulator) to detector, and (u_x, u_y) is the displacement of the pattern in units of the effective pixel size p_{eff}.

In single-shot X-ray speckle-tracking, small window patches from the sample and the reference scan are compared to calculate its displacement reaching a resolution in the range of the average speckle size [6]. Various techniques allow increasing resolution and sensitivity by stepping the modulator [6]. UMPA is a flexible processing method that allows using different numbers of

phase steps N and enables to find a good trade-off between resolution and phase sensitivity by adjusting the analysis window size w [7]. A statistical analysis of the underlying principle yields the following expression for the variance of the differential phase image [10]:

$$\sigma_x^2 = \frac{2\sigma_{\text{ph}}^2}{\sum \left|\frac{\partial I}{\partial x}\right|^2} = \frac{2\sigma_{\text{ph}}^2}{N\left\langle \left|\frac{\partial I}{\partial x}\right|^2 \right\rangle}, \tag{S7}$$

where σ_{ph}^2 is the variance of the photon noise, N is the number of pixels in a window (in case of single-shot speckle tracking like in [10]) or the number of phase steps. $\langle ... \rangle$ denotes the mean in a window or a pixel's phase stepping data set. Hence, an ideal speckle pattern should have strong intensity gradients i.e. small speckle sizes and high contrast. In the case of a periodic modulator, this is achieved at a high visibility-to-period ratio. Small periods allow a denser sampling and therefore less steps to achieve a certain resolution while high visibility reduces the noise in the phase signal. Hence, the visibility-to-period ratio (depicted by dashed lines in Figure 2(d) of the main article) is a useful estimate to compare such setups.

Figure S5 (a) and (b) show a comparison of partial derivatives in $x-$ and $y-$direction for the P1000 and the TAI 10-15 modulators (same data set as the visibility comparison in Figure 3 of the main article). The TAI modulation has both a stronger spatial gradient density as well as greater absolute gradient values. To compare the discussed modulators, we define a quantity $m = \left\langle \left|\frac{\partial I}{\partial x}\right|^2 \right\rangle^{-1}$ which scales linearly with σ_x^2 and can be calculated pixel-wise for any given intensity modulation and stepping scheme. Figure S5 (c) shows histograms of m values calculated for two TAIs and the P1000 diffuser from a ROI of 500 × 500 pixels. Both TAIs reach significantly lower m values with a much narrower distribution compared to P1000. The mean of the P1000 (1.49×10^{-4}) is more than 6 times higher than that of TAI 10-15 (0.246×10^{-4}). Since $\sigma_{\text{ph}}^2 \propto I$ [10] using the TAI instead of P1000 allows to reduce the dose and exposure time by a factor of 6 to reach comparable σ_x. Furthermore, Figure S5 (c) shows that performing much more steps (e.g. 40) with the P1000 diffuser will reduce m slightly and decrease the spread, however, will not reach comparable average gradients as the TAIs. Despite comparable visibility values (see Figure 3(c) in the main article) the TAI with 13 steps is still 5× more dose efficient than the P1000 diffuser with 40 steps ($m = 1.24 \times 10^{-4}$). In this regard, visibility values calculated by equation S3 give a first, useful estimation of the modulator performance, but do not consider the spatial intensity gradient distribution, which is the key factor for a good phase sensitivity.

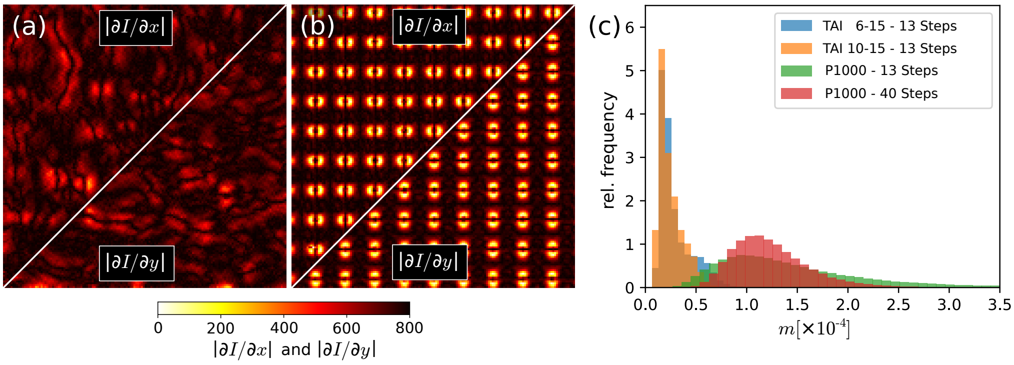

Fig. S5. Modulus gradients of the P1000 speckle pattern (a) and the TAI 10-15 modulator (b) in a 150 × 150 pixel ROI. (c) Histograms of m calculated from a 500 × 500 pixel ROI for different modulators and phase stepping scenarios.

As evident from equation S6 it is also possible to increase the propagation distance d to achieve a higher phase sensitivity. This can be easily done with the proposed TAIs by going to a higher fractional Talbot distance (e.g. to $3d_T/4$ for an increase by a factor of 4). In fact, different grating periods, duty cycles, and phase shifts provide even more flexibility to achieve optimal performance at a certain propagation distance and energy. However, too long propagation distances prove to be impractical for many samples as they result in far-field diffraction effects such as edge enhancement and deteriorate the modulation complicating phase retrieval. For some

applications, however, where smooth phase gradients are of interest (e.g. wavefront sensing, optics characterization, etc.) going to higher fractional Talbot distances is a convenient solution. It should be also noted that with increasing propagation distance the visibility drops due to limited source coherence. The projected source size in the detection plane has to be similar to the foci created by the TAI to prevent a strong visibility loss. At the same time, the modulation has to be compatible with the detector PSF to prevent further contrast loss. Using TAIs allows choosing a certain period precisely adapted to the hardware. Furthermore, they can also be adapted to certain energy and spectral range by tuning the depth of the etched trenches. Using different duty cycles also allows to operate them at different fractional Talbot distances providing more flexibility in propagation distance. In this regard, the TAIs also offer an advantage for laboratory-based systems as they reach high visibilities at short propagation distances.

A disadvantage of periodic modulators is an effect referred to as "phase wrapping" similar to grating-based imaging (GBI). Using a modulator of e.g. 10 pixels period and fitting a displacement of e.g. 4 pixels between the sample and reference scan it is not possible to distinguish from the neighboring spot which could have been shifted 6 pixels in the opposite direction. Hence, the dynamic range of the phase image is limited by the period. If a sample contains a high fraction of pixels affected by phase wrapping the range of sensitivity can be adapted by modifying d, the beam energy, or choosing a modulator with a bigger period. Furthermore, the maximum distance of the matched windows from sample and reference scan can be limited, and affected pixels can be discarded from the CT reconstruction. The same applies to sample regions where the modulation pattern is strongly deteriorated and cannot be matched reasonably to the reference pattern. This typically happens at strongly absorbing and scattering structures or at interfaces between air and soft tissue (e.g. sample vessel edges, air bubbles).

The angular sensitivity (σ_x, σ_y) reached in this work by 1D stepping was calculated from different scans performed with 13, 15, 17 and 25 steps and processed with window sizes of 3 and 5 pixels. For that the standard deviation (STD) of the signal in the differential phase images in a 150×150 pixel sample-free ROI was extracted and the sensitivity calculated by:

$$\begin{pmatrix} \sigma_x \\ \sigma_y \end{pmatrix} = \frac{p_{\text{eff}}}{d} \begin{pmatrix} \text{STD}(\text{ROI}_{\text{dx}}) \\ \text{STD}(\text{ROI}_{\text{dy}}) \end{pmatrix}. \tag{S8}$$

Here, p_{eff} is the effective pixel size. All sensitivities are tabulated in Table S3. The averaged visibility values were calculated pixel-wise from the respective flat-field steppings based on equation S3.

Table S3. Different image acquisition modes with respective visibilities and sensitivities.

Scan	N	Window [px]	Visibility	Sensitivity (σ_x/σ_y)[nrad]
Silica Particles	13	5	0.33	198 / 179
Silica Particles	15	5	0.34	179 / 176
Silica Particles	17	3	0.35	331 / 349
Silica Particles	17	5	0.35	175 / 186
Silica Particles	25	3	0.36	293 / 296
Silica Particles	25	5	0.36	153 / 156
Murine artery CT	15	3	0.42	199 / 217
Murine artery CT	15	5	0.42	95 / 107

3. ESTIMATION OF RESOLUTION

The resolution of the murine artery CT scan was estimated with two methods. First, we evaluated sharp edges in the CT slices as exemplarily shown in Figure S6(a) by the orange line and depicted by blue arrows. Here the contrast between the paraffin matrix in which the artery was embedded and the vascular wall or the lamellar structures of the tunica media is of interest. For quantitative analysis, we implemented an automated edge detection algorithm. First, pixels with steep

gradients of δ were detected by taking the spatial derivative of the image in the x-direction and applying a threshold filter. Then respective pixel values of the edges were automatically extracted and fitted by a Gauss error function as illustrated in Figure S6(b). Following criteria had to be present to select a clear edge: (1) The contrast $\Delta\delta_c$ of the edge had to be at least 0.3×10^{-7}, which is more than 20× higher than the noise level of the background paraffin wax matrix ($\sigma_N = 0.015 \times 10^{-7}$). (2) The edge had to consist of a number of pixels covering $\pm 3\sigma$ around the mean μ and (3) the residuum (measurement-fit) of every pixel value had to be less than 5% of the contrast $\Delta\delta_c$ to exclude badly shaped edges e.g. with pixel outliers. The algorithm was performed in 20 equidistant slices all over the CT volume of interest and from every slice 10 edges with the lowest σ values were extracted. From those 200 valid edges an average of $\sigma = 1.43 \pm 0.088$ pixels was found resulting in an average FWHM of 3.06 ± 0.19 µm which we consider as the spatial resolution of the scan. The second method based on the Fourier power spectrum (FPS) was proposed in [11]. A ROI (see Figure S6(a)) covering some structure of the vessel as well as the paraffin wax matrix was chosen for further analysis. The FPS was calculated according to [11], azimuthally averaged, and plotted in Figure S6(c). The noise level depicted by the dashed line was determined from the average of the last 10 frequency bins and the doubled noise level is plotted as the orange line. To determine its intersection with the FPS the latter was fitted with a 2nd order polynomial around the region of interest indicated by the red line (fit) with the respective black crossbars. The intersection depicted by the green line lies at 368 lp/mm and corresponds to a feature size of 2.7 µm.

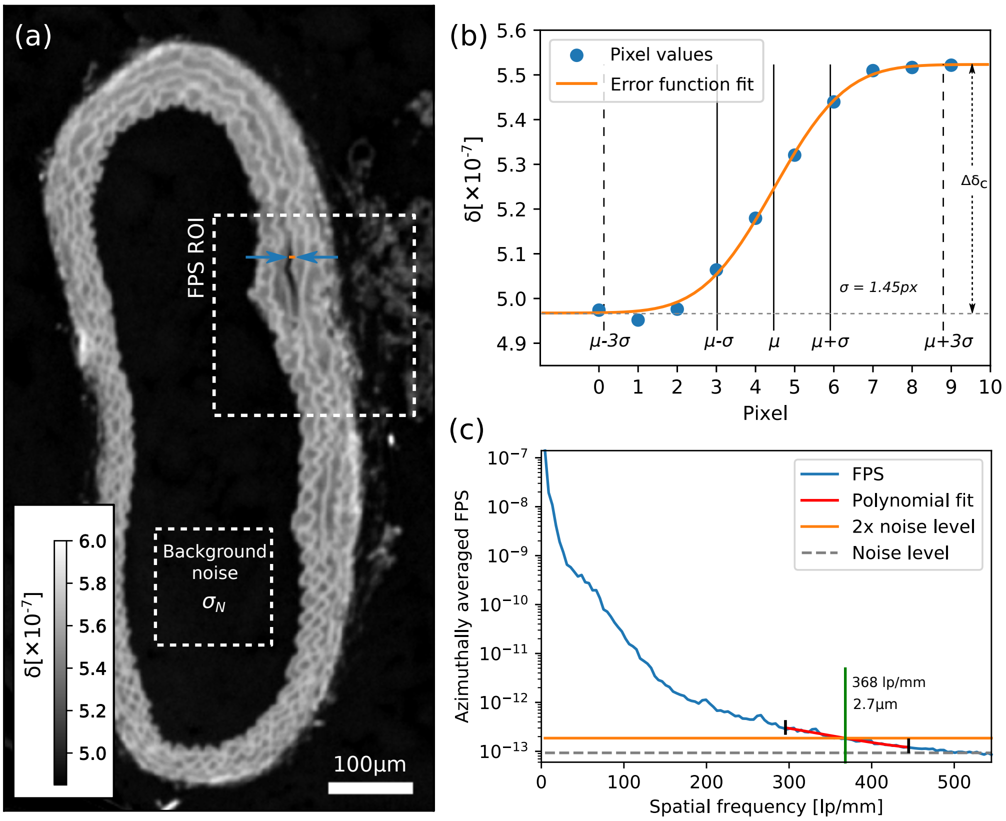

Fig. S6. (a) CT slice of the murine artery showing the ROI used for the FPS analysis, the position of an exemplary edge, and the ROI used for background noise determination. (b) The exemplary edge profile from the respective line plot in (a) fitted by the Gauss error function with pixel values in the range of at least $\pm 3\sigma$ around the mean μ. (c) Plot of the azimuthally averaged FPS from the respective ROI in (a) showing the noise background level as well as the intersection of the FPS with the doubled noise level.

REFERENCES

1. J. T. Winthrop and C. R. Worthington, “Theory of Fresnel Images I Plane Periodic Objects in Monochromatic Light,” J. Opt. Soc. Am. **55**, 373 (1965).
2. A. Schöps, P. Vagin, and M. Tischer, “Properties of the insertion devices for petra iii and its extension,” AIP Conf. Proc. **1741**, 020019 (2016).
3. S. Lautner, C. Lenz, J. U. Hammel, J. Moosmann, M. Kühn, M. Caselle, M. Vogelgesang, A. Kopmann, and F. Beckmann, “Using SRuCT to define water transport capacity in Picea abies,” (SPIE-Intl Soc Optical Eng, 2017), p. 53.
4. M. ENGELHARDT, C. KOTTLER, O. BUNK, C. DAVID, C. SCHROER, J. BAUMANN, M. SCHUSTER, and F. PFEIFFER, “The fractional talbot effect in differential x-ray phase-contrast imaging for extended and polychromatic x-ray sources,” J. Microsc. **232**, 145–157 (2008).
5. T. Thuering and M. Stampanoni, “Performance and optimization of x-ray grating interferometry,” Philos. Transactions Royal Soc. A: Math. Phys. Eng. Sci. **372**, 20130027 (2014).
6. M.-C. Zdora, “State of the Art of X-ray Speckle-Based Phase-Contrast and Dark-Field Imaging,” J. Imaging **4**, 60 (2018).
7. M. C. Zdora, P. Thibault, T. Zhou, F. J. Koch, J. Romell, S. Sala, A. Last, C. Rau, and I. Zanette, “X-ray Phase-Contrast Imaging and Metrology through Unified Modulated Pattern Analysis,” Phys. Rev. Lett. **118**, 203903 (2017).
8. C. Kottler, C. David, F. Pfeiffer, and O. Bunk, “A two-directional approach for grating based differential phase contrast imaging using hard x-rays,” Opt. Express **15**, 1175 (2007).
9. H. Wang, R. C. Atwood, M. J. Pankhurst, Y. Kashyap, B. Cai, T. Zhou, P. D. Lee, M. Drakopoulos, and K. Sawhney, “High-energy, high-resolution, fly-scan X-ray phase tomography,” Sci. Reports **9**, 1–11 (2019).
10. T. Zhou, M.-C. Zdora, I. Zanette, J. Romell, H. M. Hertz, and A. Burvall, “Noise analysis of speckle-based x-ray phase-contrast imaging,” Opt. Lett. **41**, 5490 (2016).
11. P. Modregger, D. Lübbert, P. Schäfer, and R. Köhler, “Spatial resolution in Bragg-magnified X-ray images as determined by Fourier analysis,” Phys. Status Solidi (A) Appl. Mater. Sci. **204**, 2746–2752 (2007).
